# Mechanosensitivity of Human Oligodendrocytes

**DOI:** 10.3389/fncel.2020.00222

**Published:** 2020-07-24

**Authors:** Daniela Espinosa-Hoyos, Suzanne R. Burstein, Jaaram Cha, Tanya Jain, Madhura Nijsure, Anna Jagielska, Valentina Fossati, Krystyn J. Van Vliet

**Affiliations:** ^1^Department of Chemical Engineering, Massachusetts Institute of Technology, Cambridge, MA, United States; ^2^The New York Stem Cell Foundation Research Institute, New York, NY, United States; ^3^Department of Materials Science and Engineering, Massachusetts Institute of Technology, Cambridge, MA, United States; ^4^Critical Analytics for Manufacturing Personalized-Medicine (CAMP) Interdisciplinary Research Group, Singapore-MIT Alliance for Research and Technology (SMART) CREATE, Singapore, Singapore; ^5^Department of Biological Engineering, Massachusetts Institute of Technology, Cambridge, MA, United States

**Keywords:** mechanotransduction, mechanobiology, oligodendrocyte, induced pluripotent stem cell, human models, *in vitro*, glia

## Abstract

Oligodendrocytes produce and repair myelin, which is critical for the integrity and function of the central nervous system (CNS). Oligodendrocyte and oligodendrocyte progenitor cell (OPC) biology is modulated *in vitro* by mechanical cues within the magnitudes observed *in vivo*. In some cases, these cues are sufficient to accelerate or inhibit terminal differentiation of murine oligodendrocyte progenitors. However, our understanding of oligodendrocyte lineage mechanobiology has been restricted primarily to animal models to date, due to the inaccessibility and challenges of human oligodendrocyte cell culture. Here, we probe the mechanosensitivity of human oligodendrocyte lineage cells derived from human induced pluripotent stem cells. We target phenotypically distinct stages of the human oligodendrocyte lineage and quantify the effect of substratum stiffness on cell migration and differentiation, within the range documented *in vivo*. We find that human oligodendrocyte lineage cells exhibit mechanosensitive migration and differentiation. Further, we identify two patterns of human donor line-dependent mechanosensitive differentiation. Our findings illustrate the variation among human oligodendrocyte responses, otherwise not captured by animal models, that are important for translational research. Moreover, these findings highlight the importance of studying glia under conditions that better approximate *in vivo* mechanical cues. Despite significant progress in human oligodendrocyte derivation methodology, the extended duration, low yield, and low selectivity of human-induced pluripotent stem cell-derived oligodendrocyte protocols significantly limit the scale-up and implementation of these cells and protocols for *in vivo* and *in vitro* applications. We propose that mechanical modulation, in combination with traditional soluble and insoluble factors, provides a key avenue to address these challenges in cell production and *in vitro* analysis.

## Introduction

Glial cells play fundamental roles in homeostasis, pathology, and repair of the central nervous system (CNS). Among glia, oligodendrocytes primarily produce, maintain, and repair the myelin sheaths surrounding neuronal axons, which enable cognitive and motor abilities in vertebrate animals (Bunge, 1968; Baumann and Pham-Dinh, 2001; Zalc et al., 2008). These cells reside in dynamic and complex microenvironments. The physical properties and forces in this “niche” provide cues that can regulate behavior and cell fate (Makhija et al., 2020). Changes in this mechanical landscape are characteristic of developmental (Franze, 2013; Budday et al., 2015) and aging (Morawski et al., 2014) processes, as well as various CNS disorders that include multiple sclerosis (MS; Streitberger et al., 2012; Urbanski et al., 2019), Alzheimer’s (Murphy et al., 2011, 2016) and Parkinson’s (Lipp et al., 2013). Mechanotransduction encompasses the network of mechanisms by which mechanical stimuli are converted to biochemical signals that elicit these biological responses. In adherent and anchorage-dependent cells including but not limited to glia, these mechanisms generally include integrin activation at the cell-material interface. Engagement of the actomyosin cytoskeletal network spans the cytoplasm and facilitates the transmission of force or displacement to the nuclear membrane through signaling cascades (Ingber, 2006). In the CNS, mechanical changes and dysregulation of mechanotransduction are signatures of neurological disorders (Makhija et al., 2020) that can correlate with behavioral dysfunction (Wang et al., 2018). Given the direct role of oligodendrocytes in myelination and neuronal health, we and others seek improved understanding of how these glial cells may respond to mechanical and other cues that limit or promote repair in demyelinating diseases such as MS.

Oligodendrocytes respond to both time-invariant (static) and time-varying (dynamic) mechanical cues. This mechanosensitivity has been established in the last decade using *in vitro* murine models almost exclusively, due to the practical limitations of obtaining glial cells from human CNS tissue (recently reviewed by Makhija et al., 2020). Oligodendrocyte progenitor cell (OPC) proliferation, migration, differentiation, and maturation respond to the mechanical stiffness of the materials to which these cells adhere (Jagielska et al., 2012; Lourenço et al., 2016; Urbanski et al., 2016; Segel et al., 2019), to applied uniaxial and biaxial strain (Hernandez et al., 2016; Jagielska et al., 2017), and to physical constraints (Rosenberg et al., 2008; Lee et al., 2013; Diao et al., 2015). The propensity for oligodendrocyte engagement and wrapping of synthetic axon-like fibers with myelin basic protein (MBP)-rich membrane varies with the stiffness of those cylindrical fibers, suggesting that myelination may be modulated mechanically (Espinosa-Hoyos et al., 2018). However, a full understanding of the mechanisms by which mechanical cues moderate differentiation and myelination of oligodendrocytes is incomplete. For example, mechanical stimulation can act directly through signaling pathways that begin at the interaction between integrins and extracellular ligands (O’Meara et al., 2011; Hernandez et al., 2016; Lourenço et al., 2016; Jagielska et al., 2017; Shimizu et al., 2017; Makhija et al., 2018), but may also proceed indirectly as a result of stimulation of neighboring mechanosensitive cells such as astrocytes (Moshayedi et al., 2010, 2014; Wilson et al., 2016), neurons (Jiang et al., 2011; Grevesse et al., 2015; Koser et al., 2016) and microglia (Bollmann et al., 2015). The mechanosensitivity of oligodendrocytes may have important implications in CNS pathology, and for the development of drug and cell-based therapies for remyelination. These and other implications were reviewed recently (Makhija et al., 2020).

Recent sequencing and transcriptomics studies have revealed species-specific features that highlight the importance of studying human cells to recapitulate the pathology of CNS disorders (Miller et al., 2010; Hodge et al., 2019; Jäkel et al., 2019). Genomic differences across species are also reflected in diverging aspects of mechanotransduction. For example, differential integrin expression may explain differences in susceptibility and disease progression among non-human primate species (Byrareddy et al., 2015). In other cell types such as human cancer cell lines, differences in the type and level of integrin expression and the capacity for integrin signaling have been noted among cell donor sources (Taherian et al., 2011), suggesting that aspects of mechanotransduction may be both species-specific and human donor-specific.

Human-induced pluripotent stem cells (hiPSCs) reprogrammed from epidermal fibroblasts (Takahashi et al., 2007) have enabled the production of all major human CNS cell types, carrying the genetic information of the donors (Dolmetsch and Geschwind, 2011; Rouhani et al., 2014; Goldman and Kuypers, 2015; Carcamo-Orive et al., 2017; Elitt et al., 2018; Zheng et al., 2018). Here, we differentiated human oligodendrocytes from hiPSCs and demonstrated that human oligodendrocytes exhibit mechanosensitive migration. Human oligodendrocyte migration increased with increasing substratum stiffness, consistent with previous *in vitro* findings for rat OPCs (Jagielska et al., 2012). We analyzed the differentiation of oligodendrocytes from hiPSCs of four donors and identified donor-specific responses, otherwise not captured in murine cells. These findings support the current understanding of oligodendrocytes as mechanosensitive cells, including oligodendrocytes from human donors as demonstrated herein, with some aspects of mechanotransduction in human oligodendrocytes mirroring that of murine oligodendrocytes. However, the diverging mechanosensitive trends observed among distinct human individuals indicate a potentially important role of population heterogeneity in glial cell response. These findings may have implications in demyelinating diseases and their treatment, and support the use of more biologically representative *in vitro* platforms to study complex and uniquely human diseases and enable improved approaches to personalized medicine.

## Materials and Methods

### Subjects and Cell Lines

A total of five hiPSC lines were used in this study, derived from skin biopsies of apparently healthy, deidentified donors upon specific institutional review board approvals and informed consent (Table 1). Four hiPSC donor lines were tested for each readout (migration and differentiation). All lines were reprogrammed using the NYSCF Global Stem Cell Array^®^ with the mRNA/miRNA method (StemGent). Lines were thawed, cultured and expanded on 6-well plates coated with Matrigel (Corning, cat. no. 354277) in mTeSR1 medium (StemCell Technologies, cat. no. 05850) or StemFlex medium (Thermo Fisher Scientific, Waltham, MA, USA, cat. no. A3349401). Cells were passaged enzymatically every 3-4 days using with Stempro Accutase (Thermo Fisher Scientific, Waltham, MA, USA, cat. no. A1110501) for 5 min and re-plated in mTeSR1 medium with 10 μM ROCK Inhibitor (Y2732, Stemgent, cat. no. 04-0012) for 24 h.

**Table 1:** Cell line information.

Identifier	Cell line	Sex	Age	Type of experiment
Line 1	051121-01-MR-017	Female	52 years old	Migration
Line 2	050659-01-MR-013	Female	65 years old	Migration/Differentiation
Line 3	051106-01-MR-046	Female	57 years old	Migration/Differentiation
Line 4	050743-01-MR-023	Male	51 years old	Migration/Differentiation
Line 5	051104-01-MR-040	Female	56 years old	Differentiation

### Oligodendrocyte Differentiation Protocol

The hiPSCs were differentiated to oligodendrocytes according to our previously published protocol (Douvaras and Fossati, 2015), with some modifications. Each line was differentiated twice, once for each independent experiment. Differentiation and experiments using line 4 were carried out in two independent laboratories (see Supplementary Tables S1, S2). The hiPSCs were plated in Matrigel-coated 6-well plates at a density of 100–200 thousand cells per well and cultured in mTeSR1 or StemFlex medium in a 37°C incubator with 10 μM ROCK Inhibitor (Y27632, Stemgent, cat. no. 04-0012) for 24 h. The medium was replaced daily until colonies were approximately 100–250 μm in diameter. Differentiation was induced by replacing the medium with neural induction medium (Supplementary Table S3, day 0). On day 8, the medium was switched to N2 medium (Supplementary Tables S4, S5) and replaced daily until day 12. On day 12, cells were mechanically dissociated using either the StemPro^TM^ EZPassage^TM^ Disposable Stem Cell Passaging Tool (Thermo Fisher Scientific, Waltham, MA, USA, cat. no. 23181010), or the Corning cell lifter (Millipore Sigma, cat. no. CLS3008-100EA). Each well was split across two wells of a Corning Costar ultra-low attachment 6-well plate (Millipore Sigma, cat. no. 3471) in N2B27 medium (Supplementary Table S6). Two-thirds of the medium volume were replaced every other day until day 20. On day 20, two-thirds of the medium volume were switched to PDGF medium (Supplementary Table S7), and replaced every other day until day 30. Between day 30 and day 34, golden spheroids with a diameter between 200 and 800 μm and a brown center were picked manually with a micropipette in a microscope inside a sterile hood with laminar flow. Picked spheroids were plated in PDGF medium at a density of one per well of a 24-well Nunclon-Δ tissue culture polystyrene plate (Thermo Fisher Scientific, Waltham, MA, USA, cat. no. 142475) coated with 50 μg/ml poly-L-ornithine (PO, Millipore Sigma, cat. no. 3655) and 12 μg/ml natural mouse laminin (Thermo Fisher Scientific, Waltham, MA, USA, cat. no. 23017015). Picked spheroids were also plated on polyacrylamide hydrogels coated with PO/laminin assembled in 24-well plates, at a density of one per well, in PDGF medium. Hydrogel preparation and functionalization are described in the following sections. Spheres were allowed to attach for 24 h, and two thirds of the medium were gently replaced every other day. Migration and oligodendrocyte lineage commitment was evaluated at day 60. For assessment of maturation, the medium was switched to glial or maturation medium (Supplementary Table S8) at day 60, and evaluated at day 68. GFAP+ astrocytes and MAP2+ neurons are also present in this system through day 60–68. Microglia are not generated in this protocol.

### Hydrogel Preparation

Polyacrylamide (PAAm) hydrogel disks were prepared on glass coverslips, and assembled in 24-well plates for spheroid culture experiments. The protocol was developed from a combination of previously published methods (Tse and Engler, 2010; Jagielska et al., 2012; Syed et al., 2015). Pre-cleaned glass coverslips (12 mm, no. 2 thickness) were exposed to air plasma for 5 min to hydrophilize the surface. Coverslips were immersed in 0.1 M sodium hydroxide (Millipore Sigma) in Milli-Q water for 1 min and rinsed twice with Milli-Q water. This was followed with 5 min incubation in a solution of 50% v/v (3-aminopropyl)triethoxysilane (APTES, Millipore Sigma, cat. no. A3648) in Milli-Q water. The coverslips were washed at least three times with Milli-Q water. Treated coverslips were immersed in a solution of 0.5% v/v glutaraldehyde (Millipore Sigma, cat. no. 340855) in Milli-Q water for 30 min, rinsed three times with Milli-Q water, and air dried. Glass slides were treated with trichloro (1H, 1H, 2H, 2H-perfluorooctyl) silane (PFOCTS, Millipore Sigma, cat. no. 448931) *via* chemical vapor deposition to render them superhydrophobic and thus hydrogel-repellent. Briefly, glass slides were exposed to air plasma for 5 min and placed inside a vacuum chamber next to a shallow reservoir of 80 μl of PFOCTS. Vacuum was drawn until the first bubble formed in the PFOCTS reservoir (about 5 min), and the slides were incubated for 10 min. PFOCTS-functionalized slides were stored for up to a month.

Acrylamide (AA, 40% w/v, Millipore Sigma, cat. no. A4058) and bis-acrylamide (bis-AA, 2% w/v, Thermo Fisher Scientific, Waltham, MA, USA, cat. no. BP1404) mixtures were prepared by mixing the two components with 1× phosphate buffered saline (PBS) according to the recipes in Table 2 in batches of 5 to 10 ml, vortexed for 1 min, degassed in a vacuum chamber for 30 min, and stored at 4°C for up to a month. Ammonium persulfate (APS, 50% w/v in PBS, Millipore Sigma, cat. no. A3678) and *N*,*N*,*N*′,*N*′-Tetramethylethylenediamine (TMED, 50% v/v in PBS, Millipore Sigma, cat. no. T7024) stock solutions were prepared fresh every day. Hydrogel disks were prepared up to four at a time to reduce the risk of polymerization in the preparation tube. APS (0.5% w/v) and TMED (0.05% v/v) were gently mixed with acrylamide pre-mixes with a pipette tip avoiding bubbles, as oxygen inhibits the polymerization. A 22 μl drop of the pre-polymer mixture was pipetted unto a superhydrophobic glass slide, and covered with a functionalized coverslip (treated side down). After the corresponding reaction time (Table 2), the hydrogel was immersed in PBS for a few seconds and the coverslip was carefully lifted using tweezers. Hydrogels were stored in a PBS bath for at least 24 h to swell to equilibrium before assembling the plates.

**Table 2:** Polyacrylamide recipes and apparent Young’s elastic modulus *E*, measured using atomic force microscope (AFM)-enabled indentation.

% AA* (v/v)	% Bis-AA^‡^ (v/v)	% TMED (v/v)	% APS (w/v)	Reaction time (min)	E^†^ (kPa)
18.000	0.400	0.050	0.5	4	70.29 ± 3.03
7.000	0.100	0.050	0.5	25	9.80 ± 0.47
4.000	0.045	0.050	0.5	25	1.09 ± 0.11
4.000	0.040	0.050	0.5	25	0.41 ± 0.04
4.000	0.020	0.050	0.5	25	0.12 ± 0.01

*AA stock concentration is 40% (w/v). ^‡^Bis-AA stock concentration is 2% (w/v). ^†^ ± standard deviation, n = 3 hydrogels.

Polydimethylsiloxane (PDMS) was used to immobilize hydrogel-coverslips unto multi-well plates to ease the transportation of plates across laboratories and the plating of spheroids. Dow SYLGARD^TM^ 184 PDMS base and crosslinker were vigorously mixed with a stand mixer in 1:9 ratio for 5 min and degassed in a vacuum chamber until all visible bubbles disappeared. A small drop (~10 μl) of PDMS mixture was placed in the center of each well of a 24-well plate. A hydrogel-coverslip was placed on top of each PDMS drop allowing it to spread (gel side up). Enough PBS was placed on each hydrogel to completely cover it without spilling over the sides. The plates were wrapped in parafilm and left at room temperature overnight to allow the PDMS to polymerize and solidify. The plates were flooded with PBS and stored at 4°C for up to 2 weeks.

### Functionalization of Substrata

PAAm hydrogels were allowed to equilibrate in PBS for at least 24 h before functionalization. The surface of PAAm hydrogels were covered with sulfo-SANPAH (CovaChem, cat. no. 13414) at 0.5 mg/ml in 20 mM HEPES buffer (pH 8.0). Each hydrogel was immediately exposed to UV light (8 mm light guide with collimating adaptor, OmniCure Series 2000) for 3 min at an approximate intensity of 30 mW/cm^2^, measured near the hydrogel surface. Excess solution was removed and the hydrogels were rinsed twice with 20 mM HEPES (pH 8.0). The hydrogels were subsequently shaken in an orbital shaker at 70 rpm for 1 min (to aid removal of excess sulfo-SANPAH that can otherwise precipitate out of solution and deposit on the hydrogel surface), followed by one additional wash and incubation in a solution of 50 μg/ml PO in 20 mM HEPES buffer (pH 8.0). The hydrogels were incubated in PO overnight at 4°C and washed three times with PBS. At this point functionalization was either continued in the same laboratory or in a second laboratory, where cell experiments would be carried out. For the latter, washed plates were carefully wrapped and shipped on ice overnight. PO-coated and washed plates were incubated overnight in 12 μg/ml mouse laminin in PBS at 4°C. The hydrogels were washed 3 times with PBS and incubated with DMEM/F12 at 37°C at least 30 min before plating.

Tissue culture polystyrene control plates were incubated with 50 μg/ml PO in distilled water overnight at 37°C. The PO solution was aspirated and plates were allowed to dry. Plates were subsequently incubated in 12 μg/ml mouse laminin in DMEM/F12 for 4 h. Laminin solution was aspirated and plates were washed twice with DMEM/F12 before cell plating.

### Mechanical Characterization of Polyacrylamide Hydrogels

The apparent Young’s elastic modulus *E* of PAAm hydrogels was measured *via* load-depth indentation experiments using an atomic force microscope (AFM) (MFP-3D Bio, Asylum Research). Hydrogel disks were equilibrated in 1× PBS for at least 24 h at room temperature before measurements. All hydrogels were indented while fully immersed in 1× PBS. Hydrogels with *E* below 10 kPa were indented with a spherical borosilicate probe of diameter 20 μm mounted on a cantilever of nominal spring constant *k* = 0.03 N/m (Novascan); cantilever base velocity was 6 μm/s, and probe retraction was triggered after reaching a maximum deflection of 100–200 nm. Hydrogels with *E* above 10 kPa were indented with a spherical polystyrene probe of diameter 25 μm mounted on a cantilever of nominal spring constant *k* = 0.6 N/m (Novascan); cantilever base velocity was 6 μm/s, and probe retraction was triggered after reaching a maximum deflection of 100–200 nm. Spring constants were calibrated using the thermal noise method (Hutter and Bechhoefer, 1993). At least 10 replicate indentation load-depth responses were collected per hydrogel, and three hydrogels were indented per hydrogel preparation batch. Apparent Young’s elastic modulus *E* was calculated by fitting the Hertz model to the lower 500–750 nm, which was 30% of the loading response for hydrogels below 10 kPa, and 100% of the loading response for hydrogels above 10 kPa. Values were averaged for each hydrogel sample. *E* was reported as the arithmetic mean ± standard deviation; *n* = 3 hydrogel samples for each material stiffness.

### Immunostaining

Spheroid cultures at day 60 were pre-fixed with 2% PFA in culture medium for 8-10 min, followed by fixation with 4% PFA in PBS for 8–10 min at room temperature, and washed three times with PBS. Fixed samples were blocked with 5% goat serum and 0.1% triton in PBS for 1 h at room temperature; followed by overnight incubation with primary antibodies (Supplementary Table S9) in 5% goat serum at 4°C; washed three times with PBS and incubated with secondary antibodies (Alexa Fluor, Invitrogen) at 1:500 dilution in PBS, protected from light. After three washes with PBS, nuclei were stained with DAPI (Thermo Fisher Scientific, Waltham, MA, USA, cat. no. 62248) at 1 μg/ml in PBS. These steps were performed carefully to prevent detachment of spheroids and cells. At day 68, live spheroid cultures were incubated in glial medium containing 5% goat serum for 15 min at 37°C, followed by incubation with O4 antibody in glial medium containing 5% goat serum for 45 min at 37°C. Live spheroid cultures were washed twice with DMEM and once with PBS, before proceeding with fixation as described above.

### Image Acquisition, Analysis and Statistics

Images for quantification of migration propensity were acquired at 4× magnification and stitched using the Fiji plug-in MosaicJ. Area of migration in Figure 2B was quantified from images of DAPI-stained cells. We quantified area of migration instead of distance of migration because the spheroids, and migration from the spheroids, was not always symmetric. The inner and outer perimeter of migrating cells were drawn manually in Fiji. Analysis was conducted independently by two researchers to confirm reproducibility.

**Figure 1:**
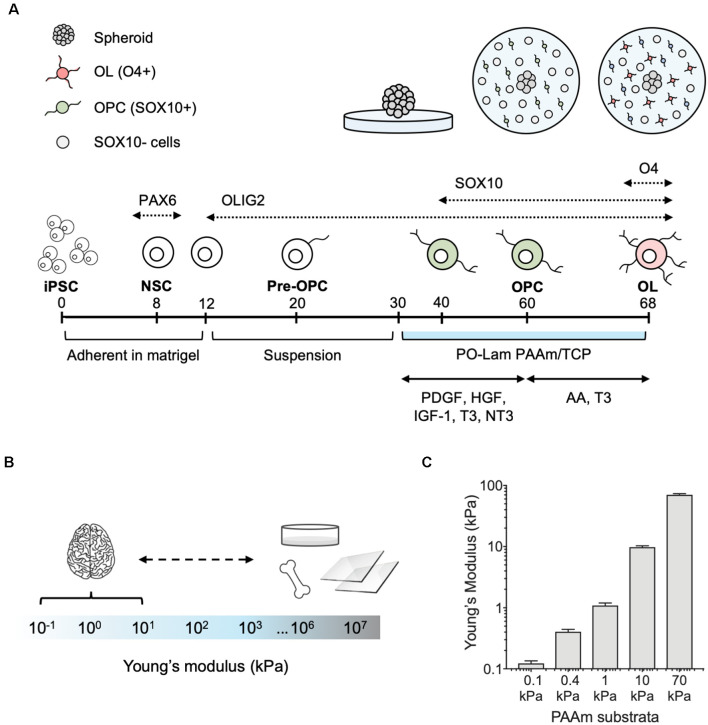
Differentiation of human oligodendrocytes from human induced pluripotent stem cells (hiPSCs) obtained from healthy donors, on compliant polyacrylamide (PAAm) hydrogels. **(A)** Human oligodendrocytes were differentiated as previously reported (Douvaras et al., 2014). At day 30 of the differentiation protocol, spheroids were plated on polyacrylamide hydrogels and tissue culture polystyrene functionalized with poly-L-ornithine and laminin. After 30 days (day 60), oligodendrocyte lineage cells were recognized by the transcription factor SOX10 (green). Migration was measured as the total surface area of the hydrogel covered by cells outside of the spheroid. Cells were grown for an additional 8 days (day 68) in maturation medium, and oligodendrocytes were stained with the O4 antibody (pink). **(B)** Range of stiffness (Young’s modulus) reported for brain tissue and typical oligodendrocyte cell culture substrata in the literature (Makhija et al., 2020). **(C)** PAAm hydrogels were prepared to span the range of physiological and pathological brain stiffness previously probed with rat primary oligodendrocytes (Jagielska et al., 2012). Mean ± standard deviation; *n* = 3 hydrogel samples for each material stiffness.

**Figure 2:**
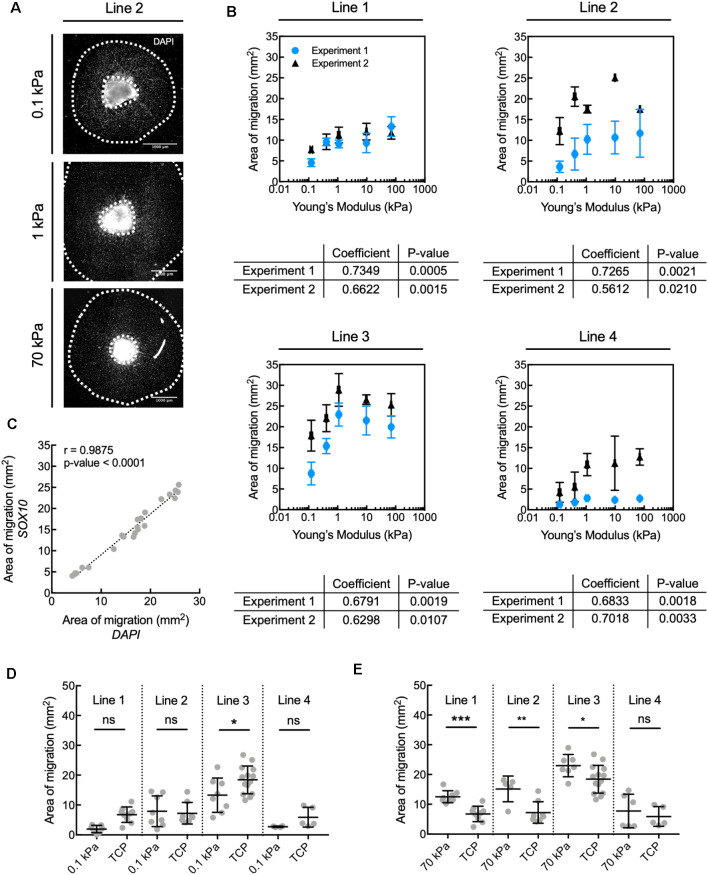
Human oligodendrocyte lineage cell migration is inhibited on compliant substrata, over the range of elastic moduli *E* = 0.1–70 kPa. **(A)** Representative images depicting spheroids (bright region in image center) and area of migration of DAPI-stained cells away from spheroids generated from donor cell line 2. Scale bars are 1 mm. **(B)** Propensity for migration of all nucleated DAPI-stained cells correlated positively with increasing stiffness of polyacrylamide hydrogel substrata, over the range of *E* = 0.1–70 kPa, for all donor cell lines. Values are mean ± standard deviation; *n* ≥ 2 spheroids per hydrogel substratum stiffness. **(A,B)** Migration from the spheroids was quantified as the total area enclosed by the outer perimeter of cells further away from the spheroid, and an inner perimeter demarcated by the edge of the spheroid. Spearman correlation coefficient was calculated across hydrogels. All coefficients were statistically significant, with *p* < 0.05. **(C)** The propensity for migration of oligodendrocyte lineage cells (SOX10+ cells) was comparable to that of the entire population (DAPI); r is the Pearson correlation coefficient; *n* = 22 spheres; pooled from two independent experiments. This can be visually appreciated in Supplementary Figure S1A. **(D)** Comparison of migration on *compliant* polyacrylamide (PAAm) hydrogels and TCP. **(E)** Comparison of migration on *stiff* polyacrylamide (PAAm) hydrogels and tissue culture polystyrene (TCP). **(D,E)** Migration was quantified as in **(B)**. Values are mean ± standard deviation; points are spheroids pooled from two independent experiments per line. Hydrogel data points are the same as in panel **(B)**. Two-tailed *t*-test, *p < 0.05, **p < 0.005 and ***p < 0.0005 for hydrogels compared with their respective TCP controls. Supplementary Figure S11 contains the graphs from panel **(B)** with adjusted axes to facilitate visual appreciation of trends across hydrogel substrata.

**Figure 3:**
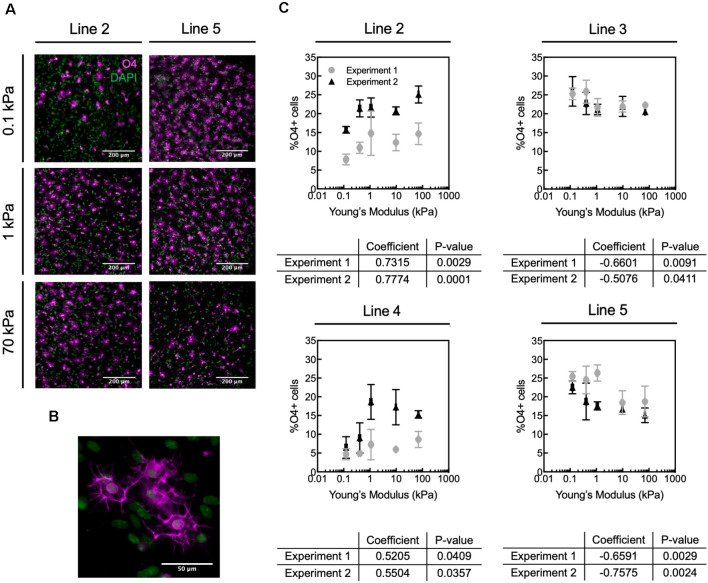
Human oligodendrocyte differentiation is modulated by substratum stiffness. **(A)** Representative images of O4+ cells (magenta) generated from two donor lines (2 and 5) that exhibited opposite mechanosensitive differentiation trends. DAPI-stained nuclei are shown in green. Scale bars are 200 μm. **(B)** Magnified image of O4+ cells. **(C)** Oligodendrocyte fraction (defined as percentage of O4+ cells) varied repeatably for a given donor with substratum stiffness, over the range of apparent Young’s elastic modulus *E* = 0.1–70 kPa. The percentage of O4+ cells was quantified as the number of O4+ cells with DAPI-stained nuclei, relative to the total number of DAPI-stained cells. However, the direction of the correlation (positive or negative correlation with stiffness) was donor dependent. Specifically, hiPSC donor lines 2 and 4 exhibited increased differentiation (% O4+ cells) with increasing material stiffness (type B response), whereas lines 3 and 5 showed decreased percentage of O4+ cells with increasing hydrogel stiffness (type A response). Data shown as mean ± standard deviation; *n* = 2–4 spheroids per stiffness condition. Spearman correlation coefficient was calculated across polyacrylamide hydrogels, and all coefficients were statistically significant with *p* < 0.05. Supplementary Figure S11 contains the graphs from panel **(C)** with adjusted axes to facilitate visual appreciation of trends across hydrogel substrata.

Images for quantification of population composition at day 60 and 68 were acquired at 10× or 20× magnification. These images were put through a custom Fiji pipeline consisting of four general steps: (1) focus correction *via* FFT bandpass filtering; (2) adaptive thresholding based on mean and standard deviation of channel intensities; (3) superposition of region positive for the marker of interest with DAPI-stained nuclei; and (4) and counting of cells with overlapping channels. The percentage of O4+ cells was quantified as the number of O4+ cells with DAPI-stained nuclei, relative to the total number of DAPI-stained cells. Mechanosensitivity trends across hydrogel substrata were evaluated with the non-parametric Spearman’s rank-order correlation coefficient, which does not assume linearity. Trends with non-zero Spearman coefficients and *p*-value < 0.05 were deemed statistically significant. The results for a given line were considered reproducible if for each independent experiment the Spearman correlation coefficient was moderate to strong (absolute magnitude higher than 0.5), had the same sign, and had a *p*-value < 0.05. Supplementary Figure S11 contains all graphs from Figures 2B, 3C with adjusted axes to facilitate visual appreciation of trends across hydrogel substrata. Tissue culture polystyrene (TCP) was not included in correlation analysis due to the inherent differences between the two material types, which are not only limited to mechanics but also encompass material chemistry. A two-tailed unpaired *t-*test was used to compare each hydrogel with its corresponding TCP controls, on spheres pooled from two replicate experiments per line. Data normality was determined with Shapiro-Wilk normality test. Statistics were performed with Prism 7 software.

## Results

### Human Oligodendrocytes Exhibit Mechanosensitive Migration *in vitro*

We adapted the directed differentiation of human oligodendrocytes from hiPSCs that we previously developed (Douvaras et al., 2014; Douvaras and Fossati, 2015) to a well-established system of polyacrylamide (PAAm) hydrogels (Tse and Engler, 2010; Pelham and Wang, 1997; Moshayedi et al., 2010; Jagielska et al., 2012) functionalized to facilitate cell adhesion to their surface (Figure 1A). The hydrogels mimic a range of stiffness described by Young’s elastic modulus *E* (of magnitude 0.1–70 kPa) that approximates and exceeds the stiffness of the human CNS environment (Murphy et al., 2016; Budday et al., 2017, 2019; McIlvain et al., 2018; Urbanski et al., 2019; Figures 1B,C). In other words, these hydrogels span the range of physiological and pathological CNS stiffness. These gels (and CNS tissue and cells) exhibit approximately six orders of magnitude lower stiffness as compared with mineralized tissue or culture plastic or glass (Figure 1B), so we refer to them as relatively compliant (i.e., low stiffness) materials. Using this same material system, we have demonstrated previously the mechanosensitivity of rat OPCs (Jagielska et al., 2012).

In the present study, we grew hiPSC-derived spheroids containing OLIG2+ progenitors from four healthy control lines (lines 1–4, Table 1) for 30 days (day 30–60) on hydrogels and standard tissue culture polystyrene or TCP (Figure 1A). The culture surfaces were functionalized with poly-L-ornithine and the integrin-specific ligand, laminin. TCP was used as the state-of-the-art control culture substratum, representing current typical *in vitro* culture conditions of oligodendrocytes and of adherent cells generally. We identified cells based on nuclear staining (DAPI). We quantified the propensity for the outward migration of cells from the spheroids as the total area enclosed by the outer perimeter of cells further away from the spheroid, and an inner perimeter demarcated by the edge of the spheroid (Figure 2A).

The propensity for migration of nucleated cells at day 60 correlated positively with the stiffness of the underlying hydrogels for *E* ranging from 0.1 kPa to 70 kPa (Figure 2B). Migration of cells positive for SOX10 (a transcription factor specific to the oligodendrocyte cell lineage) correlated strongly with the overall population migratory patterns (Figure 2C, Supplementary Figure S1A). More specifically, the propensity for migration of cells of the oligodendrocyte lineage increased with increasing substratum stiffness, with this propensity taken as the maximum distance of cell migration away from the spheroid cell source. Migration of the broader OLIG2+ population, an earlier oligodendroglia (OL) marker which also includes SOX10+ cells (Figure 1A), also correlated strongly with the migratory pattern of DAPI-stained cells (Supplementary Figures S1A,B). We further evaluated migration after 8 days of maturation (day 68), achieved by withdrawing specific growth factors such as PDGF and NT3 from the culture medium. Over these 8 days, the total cell number increased, but the correlation between substratum stiffness and area of migration was inconsistent across lines and independent experiments (Supplementary Figure S2). Results for a given line were considered inconsistent if for at least one of each independent experiment the Spearman correlation coefficient was weak (absolute magnitude lower than 0.5), had an opposite sign, or had a *p*-value > 0.05.

Given the increased efficiency of cell migration evaluated at day 60 on gels of higher stiffness over this range, we compared the extent of migration on the PAAm hydrogels to the corresponding control substratum (TCP) for each hiPSC donor line. The area of migration was similar or smaller on the most compliant hydrogels (*E* = 0.1 kPa) compared to TCP (Figure 2D). However, the area of migration was 25–85% higher on PAAm hydrogels of *E* = 70 kPa than on TCP for three out of the four lines studied (Figure 2E; migration of line 4 was statistically comparable across both types of substrata). We note that TCP differs from the PAAm hydrogels in several other characteristics besides mechanical stiffness that may affect cell adhesion and migration (Jagielska et al., 2012), and is used here to reflect typical oligodendrocyte culture conditions.

In summary, the migration of human oligodendrocyte lineage cells was modulated mechanically on PAAm hydrogels within the range of stiffness of 0.1–70 kPa. The trends were reproducible over independent experiments for a given iPSC line conducted by different researchers and laboratories (details in Supplementary Table S1). Cell migration was overall more efficient on PAAm hydrogels of *E* = 70 kPa than on TCP, the current state-of-art material, facilitating a relatively greater number of human glial cells migrating away from the spheroid of origin at a fixed time point.

### Human Oligodendrocytes Exhibit Donor-Specific, Mechanosensitive Differentiation *in vitro*

We allowed each spheroid culture from four different healthy hiPSC donor control lines (lines 2–5, Table 1) to mature separately until day 68 on hydrogels or TCP (Figure 1A). We identified cells based on nuclear staining (DAPI) and late OPCs/immature oligodendrocytes by O4 antibody staining (Figures 3A,B). We quantified the fraction of oligodendrocytes as the percentage of O4+ cells, by immunofluorescence analysis (Figure 3C). The fraction of O4+ cells was correlated with substratum stiffness for Young’s elastic moduli *E* ranging 0.1–70 kPa, across all cell lines. That is, all cell lines exhibited a mechanosensitive differentiation trend that was reproducible for a given line across two independent, replicate experiments. See Supplementary Figure S3 for differentiation efficiency on control TCP.

**Figure 4:**
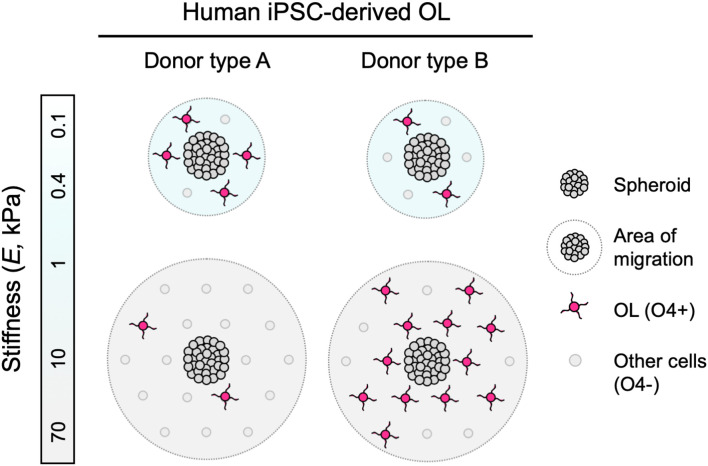
Schematic representation of findings. Human iPSC-derived oligodendroglia (OL) migrated further away from the edge of the spheroids when grown on stiffer hydrogel substrata (apparent Young’s elastic modulus or stiffness, *E* = 0.1–70 kPa), consistent for all donor lines studied. Some hiPSC donor lines, identified as type A, exhibited diminished differentiation, or lower fraction of O4+ cells, with increasing substratum stiffness. Conversely, type B lines exhibited enhanced differentiation r greater fraction of O4+ cells with increasing substratum stiffness.

We identified two types of mechanosensitive differentiation responses among the four lines studied, which we refer to hereafter as type A and type B for simplicity (Figure 4). In type A response, the fraction of O4+ cells generated *decreased* with substratum stiffness (donor lines 3 and 5). In contrast, in type B response the fraction of O4+ cells *increased* with substratum stiffness (donor lines 2 and 4). The association between expression of transcription factor OLIG2 with substratum stiffness at the end of the differentiation protocol (d68) was inconsistent (Supplementary Figure S4). Results for a given line were considered inconsistent if for at least one of each independent experiment the Spearman correlation coefficient was weak (absolute magnitude lower than 0.5), had an opposite sign, or had a *p*-value > 0.05. Thus, we did not determine OLIG2 expression to be conclusively mechanosensitive.

These results suggest the potential for donor-dependent differences in mechanosensitivity: cells obtained from different hiPSC donors under the same mechanical cues and the same chemical induction of differentiation along the oligodendrocyte lineage can respond differently. For a given donor iPSC line, these trends in stimuli response were reproducible over independent experiments conducted by different researchers and laboratories (details in Supplementary Table S2).

## Discussion

### Human Oligodendrocyte Migration Is Mechanosensitive

During mammalian development, OPCs migrate along neuronal axons and blood vessels from the neural tube to the brain and spinal cord (Gansmuller et al., 1991; Pringle and Richardson, 1993; Tsai et al., 2016). In diseases such as MS, OPCs are activated to a regenerative phenotype and migrate to active white matter lesions (Carroll and Jennings, 1994; Franklin et al., 1997; Nait-Oumesmar et al., 1999; Aguirre et al., 2007). Oligodendrocytes are anchorage-dependent cells, and their migration depends in part on integrin-mediated, dynamic, and direct interaction with the extracellular environment (Milner et al., 1996; O’Meara et al., 2016). We have demonstrated previously in a rat *in vitro* model that the mechanical properties of the environment can be permissive or inhibitory to these interactions (Jagielska et al., 2012). However, much less is known about the response of human oligodendrocytes in the context of mechanosensitive cues *in vitro* or *in vivo*. In this study, we determined that the migration of human iPSC-derived oligodendrocyte lineage cells was correlated positively with the stiffness of the underlying material substratum, consistently across the four lines studied (Figure 4). This behavior was consistent with the response of rat cells grown on materials of similar stiffness range (Young’s elastic modulus *E* = 0.1–70 kPa) and polymer chemistry (polyacrylamide), but of different ligand coating (poly-L-lysine with rat cells, poly-L-ornithine/laminin with human cells), suggesting the involvement of more than one mechanism.

Several physical parameters of spheroids have been used to predict a spheroid’s oligodendrocyte production potential qualitatively (Douvaras and Fossati, 2015). We thus explored the possibility that the size of spheroids used in our experiments could explain the quantified effect of hydrogel stiffness on the migration of oligodendrocyte lineage cells. However, we found no statistically significant correlation between the initial projected spheroid area (a measure of spheroid size, evaluated here at day 30–36) and the area of migration of nucleated cells evaluated at day 60, within the range of spheroid sizes used in these experiments (Supplementary Figure S5).

The correlation between cell migration and substratum stiffness weakened after an additional 8 days in the oligodendrocyte maturation medium (day 68, Supplementary Figure S2). Notably, reduced migration of oligodendrocyte lineage cells in maturation medium (absent growth factors PDGF, IGF, HGF, and NT3) is consistent with phenotypic maturation and onset of terminal differentiation (Noble et al., 1988; Small et al., 1988). However, GFAP+ astrocytes and MAP2+ neurons present in the culture around day 68 (Douvaras et al., 2014; Supplementary Figure S6) may have affected the measured area of migration. We note that while these methods of OPC generation from hiPSCs are among current best practices to obtain early progenitors, cells with markers of other phenotypes remain present, and efficient positive/negative selection remains work in progress.

### Oligodendrocyte Differentiation Is Mechanosensitive Across Species and Human Donor Line-Specific

We identified mechanosensitive differentiation of human oligodendrocytes derived from four hiPSC lines, reproducible over multiple independent experiments at different research sites (details in Supplementary Table S2). We quantified differentiation progression by O4 staining, a marker of pre-myelinating oligodendrocytes. We identified two types of behavior (Figure 4). Type A lines produced a lower fraction of O4+ cells with increasing substratum stiffness. Type B lines produced a higher fraction of O4+ cells with increasing substratum stiffness. The average numbers of cells across frames and conditions analyzed for each independent experiment were statistically similar (Supplementary Figure S7), and the pooling of data from independent experiments did not change the direction or statistical significance of the trends (Supplementary Figure S8). Analysis of four donor lines does not afford sufficient statistical power to conclude on the causes of population differences. However, the reproducibility of trends for a given line, particularly given the challenge and complexity of these experiments and the reproducibility obtained across different experimental research sites and researchers, supports future investigations of this divergence in donor response.

The behavior of type B lines closely matched the type of mechanoresponse observed in prior experiments using rat-derived OPCs (Jagielska et al., 2012), albeit with key differences in experimental design and initial cell composition. Mechanosensitive differentiation of murine oligodendrocytes from OPCs has been demonstrated by multiple studies (Jagielska et al., 2012; Lourenço et al., 2016; Urbanski et al., 2016; Segel et al., 2019). However, the strength and direction of reported mechanosensitive trends in response to substratum stiffness vary across studies due to multiple factors which have been reviewed recently (Makhija et al., 2020). Some of these factors include material chemistry, the type of ligand coating, and the magnitude of the mechanical cue studied. In the present study, we replicated the polyacrylamide system used in our previous work with rat primary oligodendrocytes, within the same range of Young’s elastic modulus *E* (0.1–70 kPa), for consistency. For this substratum, we previously observed increasing differentiation of primary rat oligodendrocytes (defined as % of MBP+ cells) with substratum stiffness (Jagielska et al., 2012). However, the ligand type was different (poly-D-lysine), and the metric of differentiation or maturation was the expression of MBP. While rat cells attach and grow reproducibly on substrata coated with poly-D-lysine or laminin alone, the optimal coating for hiPSC-derived spheroids was identified as poly-L-ornithine and laminin when we developed the protocol to generate oligodendrocytes on TCP (Douvaras and Fossati, 2015). To verify whether this type of ligand differentially affected the mechanoresponse of rat primary oligodendrocytes, we grew those cells on polyacrylamide hydrogels functionalized with poly-L-ornithine and laminin (the same functionalization reported herein for hiPSC-derived cells). MBP expression was positively correlated with substratum stiffness (Supplementary Figure S9). Furthermore, in the rat cell cultures, the O4+ area followed a similar correlation with substratum stiffness as the MBP+ area (Supplementary Figure S9). That is, mechanosensitive differentiation of rat oligodendrocytes can be evaluated by either O4 or MBP staining, and mechanosensitive trends identified *via* O4 staining are retained at the later stage of MBP expression. We thus infer, but do not demonstrate, that human cell lineages that exhibit mechanosensitive O4 expression will also exhibit mechanosensitive MBP expression as maturation progresses.

There are several possible explanations for heterogeneity of behavior of hiPSC lines and their derivatives. Key sources of heterogeneity include the genetic background of the hiPSC donors and batch-to-batch variations among cells obtained from the same hiPSC donor during manual steps of multi-month differentiation procedures (Rouhani et al., 2014; Morrison et al., 2016; Carcamo-Orive et al., 2017). Age and sex of cell donors can influence stem cell fate and may be explanatory variables in the behavior of derived cells (Siegel et al., 2013). The donors in this study were of similar age (52–62), and all but one (line 4) were female (Table 1). However, with four lines we do not have the statistical power to make any claims regarding the contribution of age and sex to our results. This will be interesting to consider as we expand the number of lines in future studies. Our donor lines were generated using a fully automated reprogramming platform, which was shown to significantly decrease inter-line variability compared to manual reprogramming methods (Paull et al., 2015). This suggests that the differences observed in the present study are due more plausibly to inherent genotypic differences rather than technical variations. Further, we noted the presence of cells with other phenotypic markers in these hiPSC-derived oligodendrocyte cultures (Supplementary Figure S6), a current feature of our established protocol (Douvaras et al., 2014). However, the capacity to reproduce the same experimental trends in oligodendrocyte response for a given donor across replicate experiments at different sites–using spheroids generated in different batches for that same donor line–suggests that the cell population variation in each experiment is not a dominant driver of these observations of donor-specific mechanosensitivity. Instead, we propose that genetic differences among donors may translate to differential cellular sensitivity to extracellular cues, including mechanical cues. For example, human cancer cell lines differ in their integrin expression and signaling (Taherian et al., 2011); integrin expression may differ among hiPSC-derived cell lineages sourced from different reprogrammed donor lines. Integrins play a prominent role in mechanotransduction (Ingber, 2006) and the oligodendrocyte bio machinery (Milner et al., 1996; Frost et al., 1999; O’Meara et al., 2011). Blocking of integrins inhibits migration, and the orchestrated regulation of integrin subtypes accompanies the switch from a migratory to a differentiating phenotype (Milner et al., 1996). Extracellular biochemical signals in CNS pathological conditions such as Alzheimer’s disease can directly target integrin receptors and affect the oligodendrocyte phenotype and oligodendrocyte response to injury (Quintela-López et al., 2019). Furthermore, other cell surface receptors such as cadherins further mediate cell-cell interactions in healthy and diseased environments (Payne et al., 1996; Schnädelbach et al., 2000; Ingber, 2006; Chen et al., 2017). Differential expression of these surface receptors among hiPSC donors may be reflected in a range of responses to mechanical stimulation, affect the way or extent to which oligodendrocytes interact with neighboring neurons and glia, and modulate the effect of repurposed promyelination compounds which may target some of these mechanotransduction pathways. Elucidating these differences among hiPSC lines and donors may help rationalize the spectrum of responses and lead to new or personalized targets for therapeutic intervention.

The human cell culture system studied herein is more complex than previous murine systems, which usually consist of highly enriched oligodendrocyte progenitors obtained from neonatal CNS tissue. Interactions between different CNS cell types, which can occur in hiPSC culture expansions under current protocols, may affect oligodendrocyte behavior *in vitro* and *in vivo* (Domingues et al., 2016). Mechanical stimulation also affects astrocyte and neuron behavior *in vitro*, at least for cells obtained from animal sources (Moshayedi et al., 2010, 2014; Jiang et al., 2011; Grevesse et al., 2015; Chen et al., 2016; Wilson et al., 2016). Thus, mechanical stimulation in our hiPSC-derived cell culture system may have direct and indirect effects on the behavior of oligodendrocyte-lineage cells within the cultured cell population. While such complexity is more representative of *in vivo* complexity, it is not always desirable for fundamental studies of human oligodendrocyte biology and may be ameliorated by improved differentiation and cell purification methods (Lee et al., 2014). Until such advances are demonstrated reproducibly, it is important to be mindful of this challenge when investigating the mechanobiology of hiPSC-derived oligodendrocytes. The contribution of cell-cell interactions will be crucial to consider as the field moves toward understanding the prevalence of mechanotransduction in homeostasis and disease of the CNS.

### Implications of Human Oligodendrocyte Mechanotransduction

This study suggests several implications for understanding human oligodendrocyte biology and its application to disease treatment. Certainly, additional studies are necessary to distinguish correlation and cause. Figure 4 illustrates consistent mechanosensitive migration of oligodendrocyte lineage cells, and two types of differentiation trends, based on the patterns of mechanosensitivity that we observed across the cell lines tested. Because our hiPSC lines were generated with a robotic platform, technical variations due to manual handling during the process were eliminated, and differences between the lines are attributed primarily to donor-to-donor genotypic differences. We note that while we screened only four lines for the phenotypic maturation to oligodendrocyte lineage cells, we found reproducible differences among those healthy donor cell sources. However, donor type A and type B classifications currently only describe the human cell lines herein studied, and are by no means intended to generalize patterns of human oligodendrocyte behavior. Instead, our findings suggest that these differences are worth investigating in a larger number of hiPSC lines, in both healthy and disease contexts, wherein the prevalence of these patterns may be explored, and new patterns may be uncovered. Such studies may enable understanding of how the genetic background and environmental milieu interact to determine donor-specific mechanosensitive responses and how this interaction may be altered in disease settings such as in a lesioned brain region.

We also found that contrary to rat primary oligodendrocytes grown in a similar material system (Jagielska et al., 2012), the human oligodendrocyte differentiation of type A lines was enhanced on a more compliant substrate of similar chemical composition, within the range of *E* = 0.1–70 kPa (Figure 4). It is possible that these differences reflect differences among species in oligodendrocyte differentiation, or that they reflect certain artifacts of the hiPSC-derived oligodendrocyte lineage cells (for example, cell identity purity). A shift toward wider use of human oligodendrocytes is warranted so that the community may consider whether and when studies using rodent-derived cells are predictive of human cell responses *in vitro* or *in vivo*.

Little is known about how forces and mechanoresponses measured *in vitro* on simplified materials relate to forces and responses in living cells and tissues. Techniques have been developed to measure force-displacement responses and mechanical properties of living tissues, for example, human skin (Flynn et al., 2011) and mouse brain (Thompson et al., 2019). Mechanotransduction has been studied *in vivo* in smaller organisms at different length scales. Specifically, the effect of macroscopic mechanical stimulation of whole organisms such as drosophila have been linked to changes in protein expression (Desprat et al., 2008). In the developing Xenopus brain, patterns of changing tissue stiffness appear to guide the growth direction of neuronal axons (Thompson et al., 2019). At the molecular scale, stretching of the adhesion protein talin that links integrins in the cell membrane to the actin cytoskeleton was mediated by myosin contraction of actin filaments, and suggests that adhesion proteins also play a role in mechanotransduction *in vivo* (Margadant et al., 2011). Dysregulation of mechanotransduction signaling in CNS disorders has also been linked to behavioral dysfunction in *in vivo* models (Wang et al., 2018). Understanding the prevalence of oligodendrocyte mechanotransduction *in vivo*, and the magnitude of *in vivo* mechanoresponses, at least in animal models, will help elucidate the relevance and true implications of *in vitro* studies of oligodendrocyte mechanosensitivity, and better inform the design of future translatable studies. The experimental design for such *in vivo* animal studies is not trivial and need to be evaluated carefully. Perturbation of mechanical properties of the extracellular matrix or other components of the niche with which oligodendrocytes may interact, such as neuronal axons, can have a myriad of side effects that may also influence oligodendrocyte biology, and would be challenging to decouple. Relating the results of such *in vivo* animal studies to *in vivo* human biomechanics is more complicated. Until better and more accessible human or humanized *ex vivo* or *in vivo* models are available, we will continue to rely on *in vitro* experiments with derived human cells to determine the translatability of such animal work.

Beyond these intriguing differences in mechanosensitivity of hiPSC-derived OPCs, we briefly note the increased proliferation observed on one of these hydrogels, relative to current practice. Production of human oligodendrocytes for basic and applied studies requires a stage of cell proliferation. In the present study, the extent of migration of cells was higher, or at least similar, on stiffer PAAm hydrogels (*E* = 70 kPa) compared to TCP (Figure 2E). Furthermore, oligodendrocyte lineage cells migrated as far as the other cells within that population (Figure 2C). Because the extent of migration of hiPSC-derived cells correlated positively with the number of cells that migrated outward from the spheroids (Supplementary Figure S10), migration in this system was a surrogate metric for cell production potential. Therefore, we demonstrated that overall production of human oligodendrocyte lineage cells increased by culturing hiPSC-derived spheroids on PAAm hydrogels of *E* = 70 kPa instead of standard TCP. It is important to note that while stiffness is one material property that distinguishes these hydrogels from TCP, it is certainly not the only differing characteristic of these two material types that affects cell adhesion, proliferation, migration, and differentiation. Thus, while we do not claim substratum mechanics as the only explanatory variable for the larger number of oligodendrocytes obtained on this hydrogel compared with on standard TCP, we conclude that mechanotransduction may be used in concert with other biochemical factors to modulate oligodendrocyte biology to improve processes *in vitro* such as migration or proliferation. That increased efficiency of cell proliferation may be advantageous, for example, in the advancement and viability of cell therapies.

Overall, our results show that human oligodendrocyte biology is affected by extracellular mechanics, and emphasizes the role of mechanosensing in phenotypic maturation and migration of cells comprising the CNS. This work highlights the importance of studying CNS stem and glial cells under *in vitro* conditions that more closely approximate *in vivo* cues and environments. Understanding what makes cells from healthy or diseased cohorts respond poorly in terms of migration or differentiation in a CNS tissue lesion environment can offer new directions for treatment. Additionally, studying the pathways affected by such mechanical stimulation may offer new targets for intervention, including for approaches to cell therapy and personalized medicine.

## Data Availability Statement

All datasets presented in this study are included in the article/Supplementary Material.

## Author Contributions

DE-H co-designed the study, carried out material synthesis and characterization, cell experiments and analysis, drafted and edited the manuscript, and created and edited the figures. SRB carried out cell experiments, reviewed all results, and edited the manuscript and figures. JC carried out cell experiments and analysis. TJ co-designed the study, carried out cell experiments and edited the manuscript and figures. MN carried out cell experiments and edited the manuscript and figures. AJ co-designed the study, reviewed all results and edited the manuscript and figures. VF and KJVV co-led the research team, co-designed the study, reviewed all results, and edited the manuscript and figures.

## Conflict of Interest

The authors declare that the research was conducted in the absence of any commercial or financial relationships that could be construed as a potential conflict of interest.
