# Supplementary material for: Mechanosensitivity of Human Oligodendrocytes
Source: Front Cell Neurosci. 2020 Jul 24;14:222. doi: 10.3389/fncel.2020.00222 (PMC7420028; doi:10.3389/fncel.2020.00222)
Supplement: Supplementary file 1 [file Data_Sheet_1.PDF]

Supplementary Material

Table S1. Average area of migration, acquired at day 60. Data represented as mean ± SD.

	*Line 1 (Average area of migration, mm^2)					
	**0.1 kPa**	**0.4 kPa**	**1 kPa**	**10 kPa**	**70 kPa**	**TCP**
**Experiment 1**	4.57 ± 0.95	9.58 ± 0.91	9.09 ± 0.95	9.33 ± 2.35	13.29 ± 2.38	6.08 ± 2.39
**Experiment 2**	7.72 ± 0.32	9.61 ± 1.86	11.25 ± 1.90	12.06 ± 2.01	11.77 ± 1.53	8.11 ± 2.86
	**Line 2 (Average area of migration, mm^2)					
	**0.1 kPa**	**0.4 kPa**	**1 kPa**	**10 kPa**	**70 kPa**	**TCP**
**Experiment 1**	3.64 ± 1.38	6.71 ± 3.87	10.26 ± 3.61	10.69 ± 3.94	11.70 ± 5.79	9.48 ± 4.07
**Experiment 2**	12.22 ± 3.28	20.56 ± 2.35	17.55 ± 0.922	25.09 ± 0.82	17.48 ± 0.62	5.01 ± 0.84
	†Line 3 (Average area of migration, mm^2)					
	**0.1 kPa**	**0.4 kPa**	**1 kPa**	**10 kPa**	**70 kPa**	**TCP**
**Experiment 1**	8.74 ± 2.73	15.37 ± 1.82	22.95 ± 2.78	21.50 ± 3.48	19.97 ± 2.67	15.28 ± 2.77
**Experiment 2**	17.87 ± 3.69	22.05 ± 3.26	2.90 ± 3.95	26.57 ± 1.14	25.26 ± 2.72	22.64 ± 2.84
	‡Line 4 (Average area of migration, mm^2)*					
	**0.1 kPa**	**0.4 kPa**	**1 kPa**	**10 kPa**	**70 kPa**	**TCP**
**Experiment 1**	4.22 ± 2.40	5.51 ± 3.59	10.93 ± 2.68	11.24 ± 6.53	12.77 ± 2.00	7.29 ± 3.84
**Experiment 2**	1.28 ± 0.32	1.78 ± 0.70	2.80 ± 0.51	2.38 ± 0.52	2.74 ± 0.10	3.82 ± 0.12

*Line 1: Hydrogel fabrication and functionalization were performed by the same researcher in laboratory #1; cell experiments for experiments one and two were carried out by the same researcher in laboratory #2.

**Line 2: Hydrogel fabrication and functionalization were performed by the same researcher in laboratory #1; cell experiments for experiments one and two were carried out by two different researchers in laboratory #2.

†Line 3: Hydrogel fabrication and functionalization were performed by the same researcher in laboratory #1; cell experiments for experiments one and two were carried out by the same researcher in laboratory #1.

‡Line 4: Hydrogel fabrication and functionalization were performed by two different researchers in laboratory #1; cell experiments and image acquisition for experiments one and two were carried out in laboratory #2 and laboratory #1, respectively.

Table S2. Percentage of O4+ cells, acquired at day 68. Data represented as mean ± SD.

	‡**Line 2** (% O4+ cells)					
	**0.1 kPa**	**0.4 kPa**	**1 kPa**	**10 kPa**	**70 kPa**	**TCP**
**Experiment 1**	7.84 ± 1.43	10.95 ± 1.49	14.80 ± 5.88	12.34 ± 2.18	14.67 ± 2.86	17.40 ± 0.00
**Experiment 2**	16.54 ± 0.25	22.38 ± 1.88	21.80 ± 2.64	21.00 ± 1.19	25.26 ± 0.85	18.96 ± 3.05
	†**Line 3** (% O4+ cells)					
	**0.1 kPa**	**0.4 kPa**	**1 kPa**	**10 kPa**	**70 kPa**	**TCP**
**Experiment 1**	25.27 ± 1.55	25.85 ± 3.07	21.68 ± 2.32	21.93 ± 1.46	22.26 ± 0.59	18.56 ± 0.97
**Experiment 2**	25.94 ± 3.90	27.73 ± 2.95	21.17 ± 1.37	21.94 ± 2.67	20.52 ± 0.86	25.03 ± 0.35
	***Line 4** (% O4+ cells)					
	**0.1 kPa**	**0.4 kPa**	**1 kPa**	**10 kPa**	**70 kPa**	**TCP**
**Experiment 1**	6.49 ± 2.87	9.03 ± 4.04	18.62 ± 4.65	17.22 ± 4.69	15.29 ± 0.99	7.19 ± 0.87
**Experiment 2**	4.68 ± 1.47	4.87 ± 0.00	7.23 ± 4.01	5.96 ± 0.71	8.61 ± 2.13	5.34 ± 1.58
	‡**Line 5** (% O4+ cells)					
	**0.1 kPa**	**0.4 kPa**	**1 kPa**	**10 kPa**	**70 kPa**	**TCP**
**Experiment 1**	25.54 ± 1.32	24.48 ± 3.70	26.35 ± 2.17	18.43 ± 3.16	18.73 ± 4.13	16.69 ± 2.81
**Experiment 2**	22.50 ± 1.70	18.73 ± 4.90	17.58 ± 1.09	16.67 ± 0.70	15.05 ± 1.97	18.81 ± 0.94

‡Lines 2 and 5: Hydrogel fabrication and functionalization were performed by the same researcher in laboratory #1; cell experiments for experiments one and two were carried out by two different researchers in laboratory #2.

†Line 3: Hydrogel fabrication and functionalization were performed by the same researcher in laboratory #1; cell experiments for experiments one and two were carried out by the same researcher in laboratory #1.

*Line 4: Hydrogel fabrication and functionalization were performed by two different researchers in laboratory #1; cell experiments and image acquisition for experiments one and two were carried out in laboratory #2 and laboratory #1, respectively.

Table S3. Neural induction medium composition (day 0 through day 7)

Component	Concentration	Source	Catalog No.
mTeSR Custom		StemCell Technologies	Customized mTeSR1 w/o five pluripotency factors
PenStrep (100x)	1x	Life Technologies	15070063
SB431542	10 µM	Stemgent	04-0010
LDN193189	250 nM	Stemgent	040074
Retinoic acid	100 nM	MilliporeSigma	R2625

Table S4. Basal medium composition

Component	Concentration	Source	Catalog No.
DMEM/F12		Life Technologies	11320082
PenStrep (100x)	1x	Life Technologies	15070063
GllutaMAX-I (100x)	1x	Life Technologies	04-0010
MEM non-essential amino acids (100)	1x	Life Technologies	11140-050
2-Mercaptoethanol (1000x)	1x	Life Technologies	21985023

Table S5. N2 medium composition (day 8 through day 11)

Component	Concentration	Source	Catalog No.
Basal medium (Table S4)	-	-	-
N2 supplement (100x)	1x	Life Technologies	17502-048
Retinoic acid	100 nM	MilliporeSigma	R2625

Smoothened agonist	1 μM	EMD-Millipore	566660

Table S6. N2B27 medium composition (day 12 through day 19)

Component	Concentration	Source	Catalog No.
Basal medium (Table S4)	-	-	-
N2 supplement (100x)	1x	Life Technologies	17502-048
B27 supplement w/o vitamin A (50x)	1x	Life Technologies	12587-010
Human insulin solution	25 μg/mL	MilliporeSigma	19278
Retinoic acid	100 nM	MilliporeSigma	R2625
Smoothened agonist	1 μM	EMD-Millipore	566660

Table S7. PDGF medium composition (day 20 through day 59)

N2B27 medium (d12-d19)			
**Component**	**Concentration**	**Source**	**Catalog No.**
Basal medium (Table S4)	-	-	-
N2 supplement (100x)	1x	Life Technologies	17502-048
B27 supplement w/o vitamin A (50x)	1x	Life Technologies	12587-010
Human insulin solution	25 μg/mL	MilliporeSigma	19278
Human PDGFaa	10 ng/mL	R&D Systems	221-AA-050
Human IGF-1	10 ng/mL	R&D Systems	291-G1-200
Human HGF	5 ng/mL	R&D Systems	294-HG-025

Neurotrophin 3 (NT3)	10 ng/mL	EMD-Millipore	GF031
3,3,5-Triiodo-L-thyronine (T3)	60 ng/mL	MilliporeSigma	T2877
N6,2′-O-Dibutyryladenosine 3′,5′-cyclic monophosphate sodium salt (cAMP)	1 µM	MilliporeSigma	D0260
Biotin	100 ng/mL	MilliporeSigma	4639

Table S8. Glial medium composition (day 20 through day 59)

Component	Concentration	Source	Catalog No.
Basal medium (Table S4)	-	-	-
N2 supplement (100x)	1x	Life Technologies	17502-048
B27 supplement w/o vitamin A (50x)	1x	Life Technologies	12587-010
Human insulin solution	25 µg/mL	MilliporeSigma	19278
3,3,5-Triiodo-L-thyronine (T3)	60 ng/mL	MilliporeSigma	T2877
N6,2′-O-Dibutyryladenosine 3′,5′-cyclic monophosphate sodium salt (cAMP)	1 µM	MilliporeSigma	D0260
Biotin	100 ng/mL	MilliporeSigma	4639
HEPES	10 nM	MilliporeSigma	H4034
Ascorbic acid	20 µg/mL	MilliporeSigma	A4403

Table S9. Primary antibodies used for immunocytochemistry.

Primary antibodies				
**Antigen**	**Dilution**	**Host**	**Source**	**cat no**
O4	1:30	Mouse	J.G. Laboratory	-
MBP	1:200	Rat	Abcam	ab7349
OLIG2	1:500	Rabbit	Millipore	MABN50
SOX10	1:100	Goat	R&D	SF2864
GFAP	1:750	Rabbit	Dako	ZO334001
MAP2	1:1000	Chicken	Abcam	ab5392

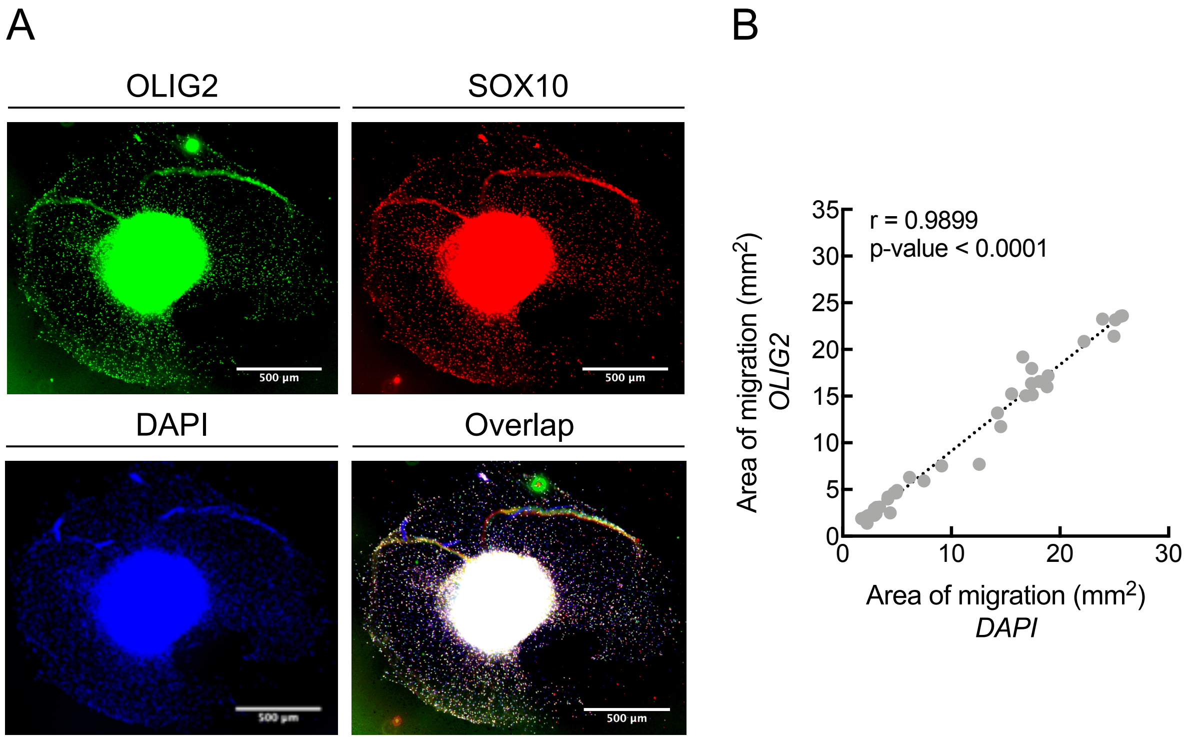

Figure S1. (A) Example of OLIG2, SOX10 and DAPI staining at day 60. (B) The propensity for migration of OLIG2+ cells was comparable to that of the entire population identified by DAPI; r is the Pearson correlation coefficient; n = 42 spheres; pooled from three independent experiments.

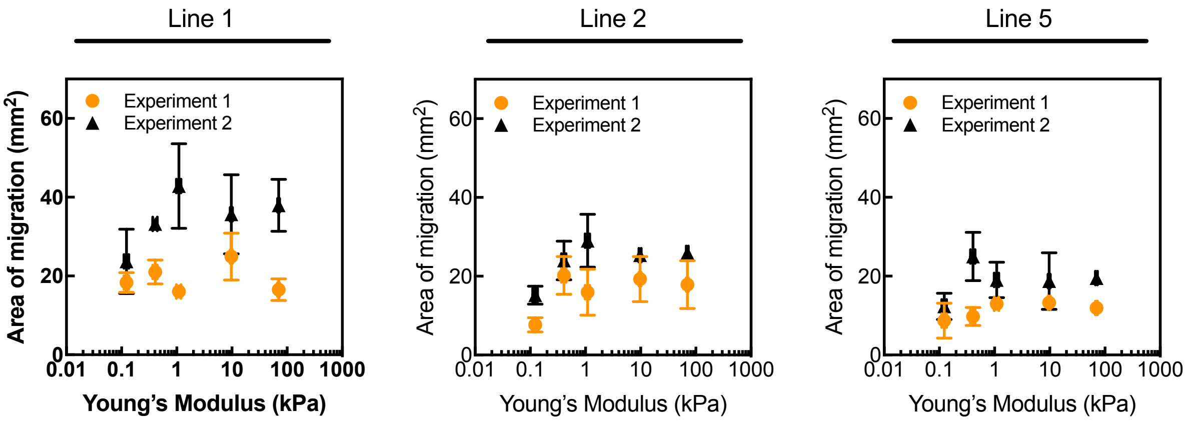

Spearman coefficients

	Line 1	Line 2	Line 5
**Experiment 1**	-0.1888	0.5085	0.4047
**Experiment 2**	0.4527	0.6026	0.2608

p-values

	Line 1	Line 2	Line 5
**Experiment 1**	0.4390	0.0312	0.0768
**Experiment 2**	0.0516	0.0120	0.3449

Figure S2. The extent of migration of cells at day 68 is not consistently correlated with substratum stiffness over the range of 0.1-70 kPa. Values are mean ± standard deviation; n ≥ 2 spheroids per stiffness. The Spearman correlation coefficient r was calculated across polyacrylamide hydrogels. Area of migration was defined as the area between the two dotted lines (see Figure 2).

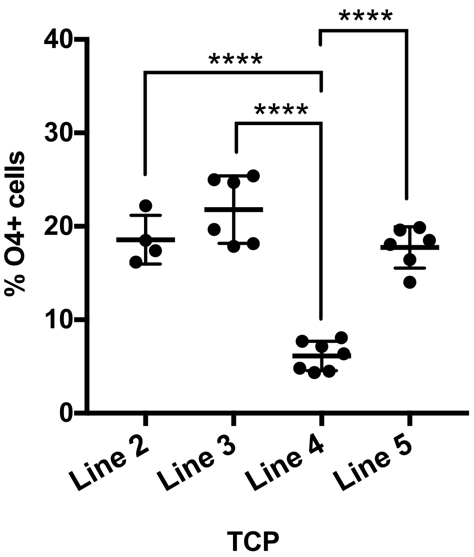

Figure S3. %O4+ cells generated on control tissue culture polystyrene (TCP), measured at day 68. Values are mean ± standard deviation; points are spheroids pooled from two independent experiments per line. One-way ANOVA with Tukey's post hoc multiple comparisons, ****p < 0.0001.

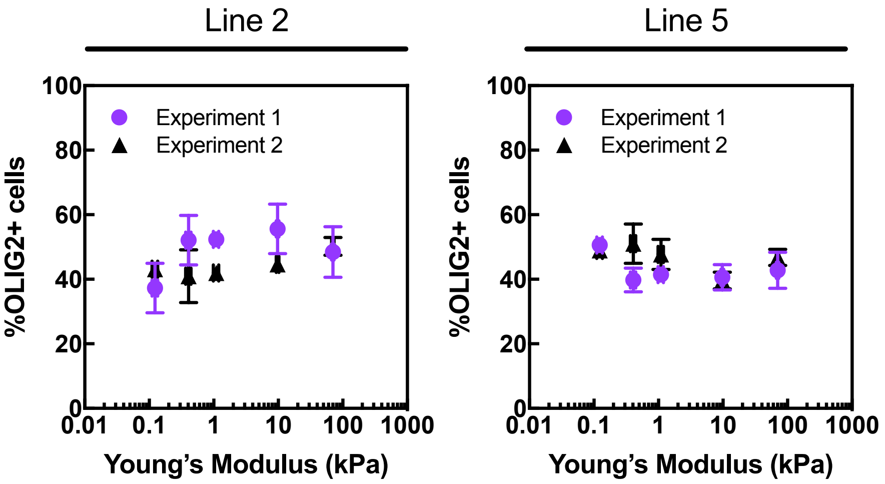

Spearman coefficients

	Line 2	Line 5
**Experiment 1**	0.3895	-0.227
**Experiment 2**	0.706	-0.4586

p-values

	Line 2	Line 5
**Experiment 1**	0.0089	0.4317
**Experiment 2**	0.0118	0.1164

Figure S4. The % of OLIG2 positive cells (early differentiation marker) is inconsistently correlated with substratum stiffness. Statistically significant correlations were in the same direction as the corresponding correlations of % of O4+ cells (Figure 3). Values are mean ± standard deviation; n = 2-4 spheroids per stiffness. The Spearman correlation coefficient was calculated across polyacrylamide hydrogels.

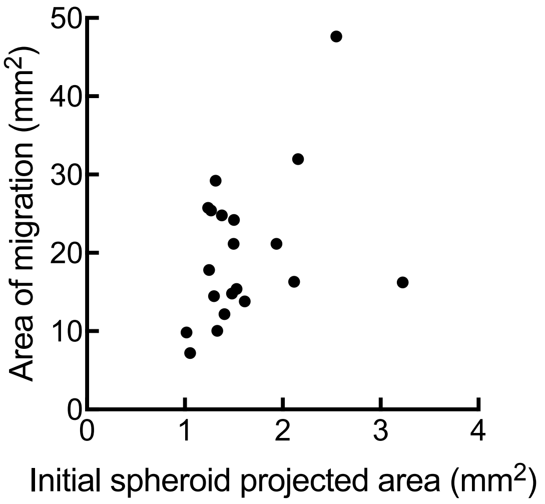

Figure S5. The initial projected size of plated spheroids at the beginning of the experiment is not correlated (Spearman correlation) with the area of migration of cells generated by the spheroids, and thus cannot explain the observed correlation between area of migration and substratum stiffness. The projected area of each spheroid was measured from images acquired after plating, around day 30-36; the area of migration of cells generated by the same spheroid was measured at day 60. Spearman r = 0.3023, p = 0.1952.

Line 51106

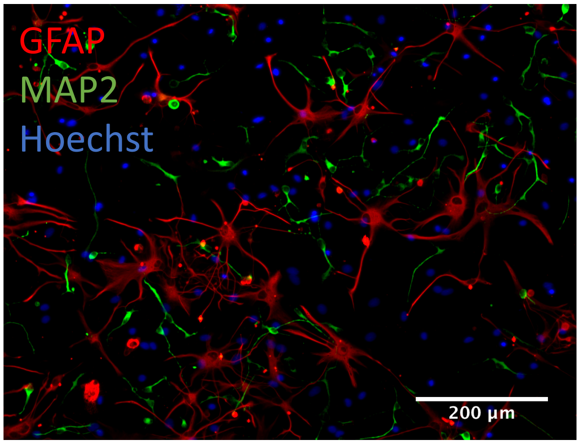

Figure S6. Example of MAP2+ neurons and GFAP+ astrocytes present in iPSC-derived oligodendrocyte cultures through days 60 and 68.

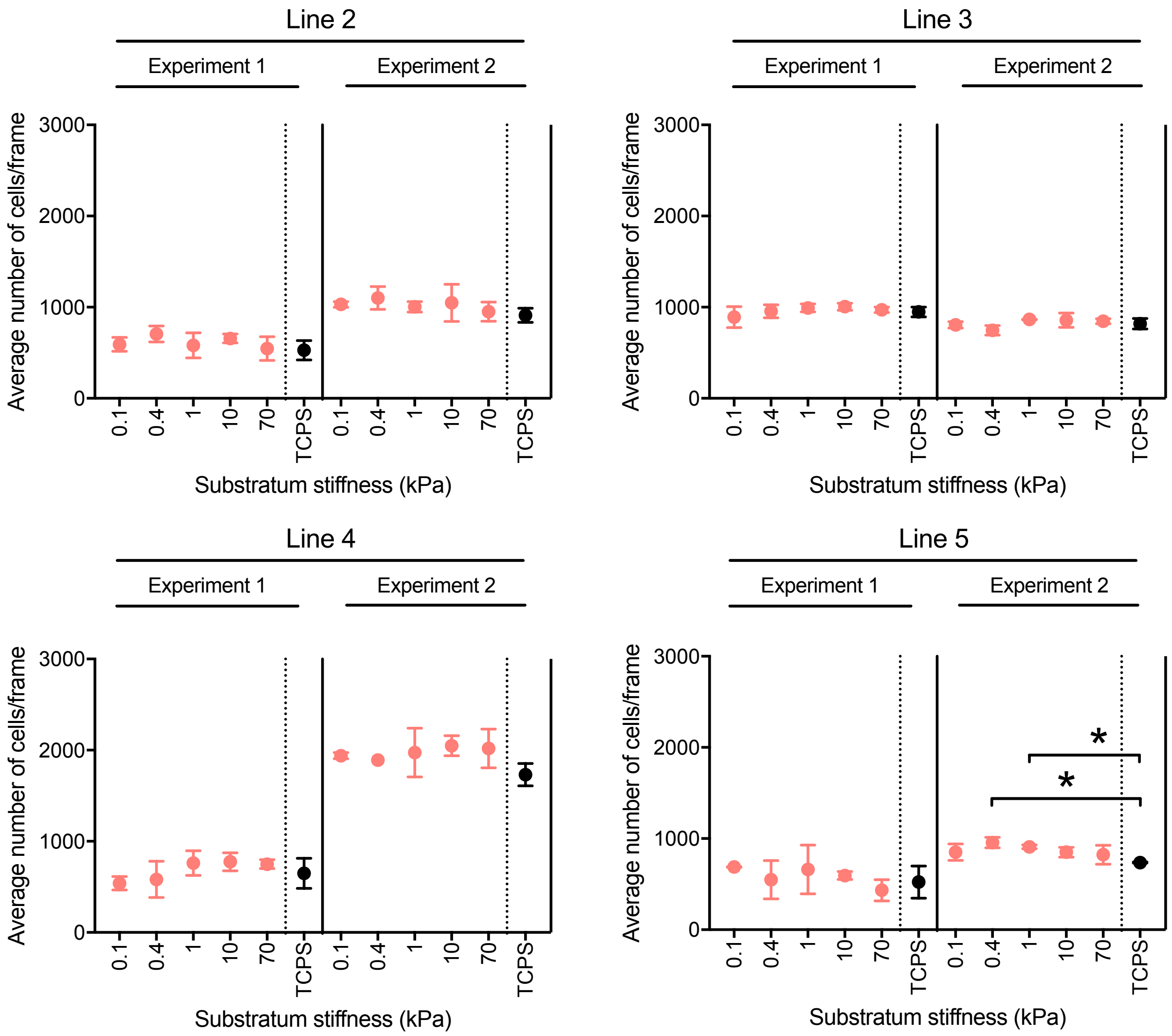

Figure S7. Cell density cannot explain the observed correlation between %O4+ cells and stiffness of PAAm hydrogels with Young's modulus in the range of 0.1 kPa and 70 kPa at day 68, or the differences between PAAm 0.1 kPa or 70 kPa with TCPS. One-way ANOVA with post-hoc Tukey's multiple comparisons , $^*p<0.05$; $n = 2\text{-}3$ wells per condition; error bars are mean ± SD.

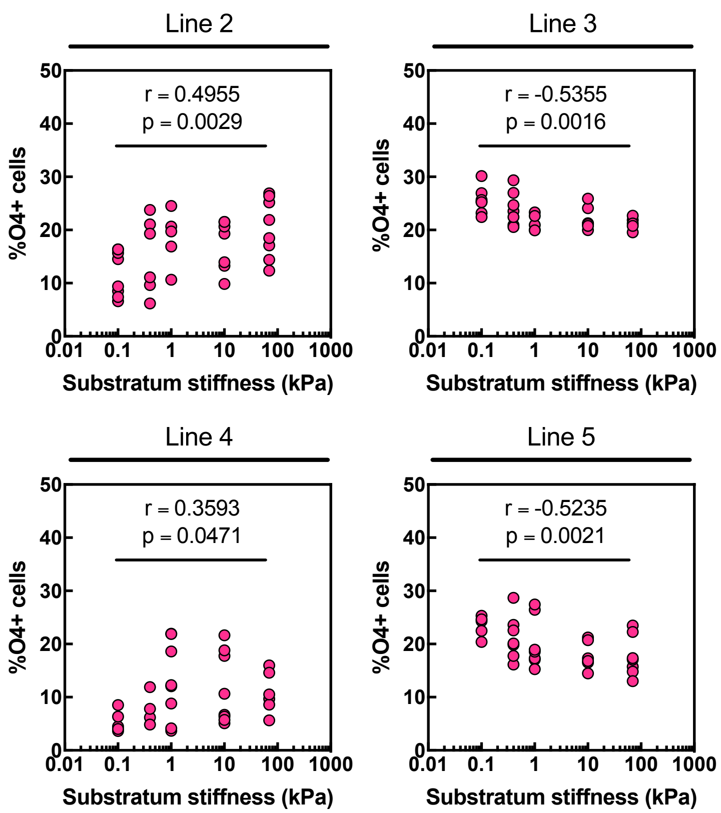

Figure S8. Statistics on combined data points of O4 data from independent experiments. Various factors account for variability in absolute magnitude of differentiation efficiencies across independent experiments. When the data from independent experiment are pooled, mechanodifferentiation trends persist, with moderate Spearman correlation coefficients (r) that are statistically significant

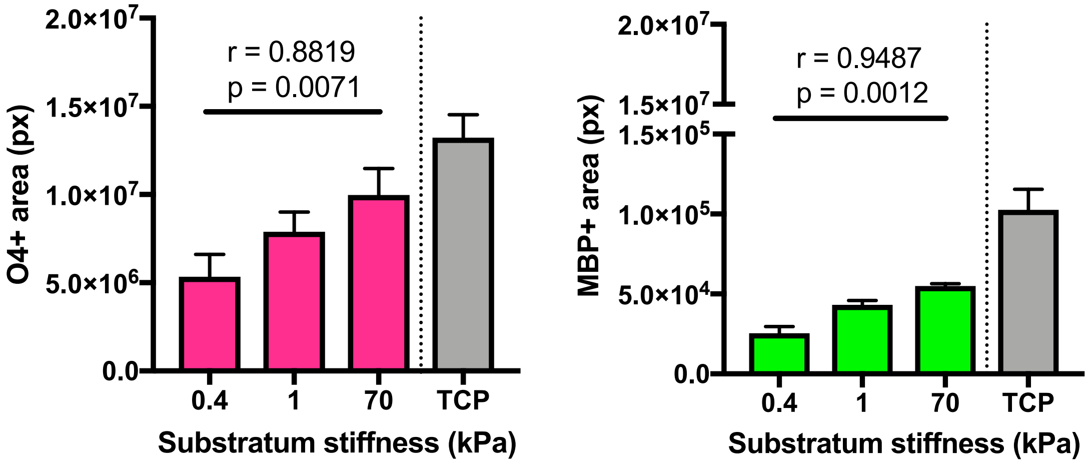

Figure S9. Rat oligodendrocyte differentiation on polyacrylamide hydrogels and standard tissue culture polystyrene (TCP) functionalized with poly-L-ornithine and laminin. Myelin basic protein expression increases with substratum stiffness, as previously demonstrated (Jagielska et al., 2012). Expression of the sulfatide recognized by O4 antibody is also positively correlated with substratum stiffness. MBP and O4 expression were assessed by immunostaining and image analysis; quantified as the total pixel area with signal above background, per frame, and averaged over at least 15 frames acquired at 20x magnification. Error bars are mean ± standard deviation across 2-3 wells, for one experiment. R is the Spearman correlation coefficient.

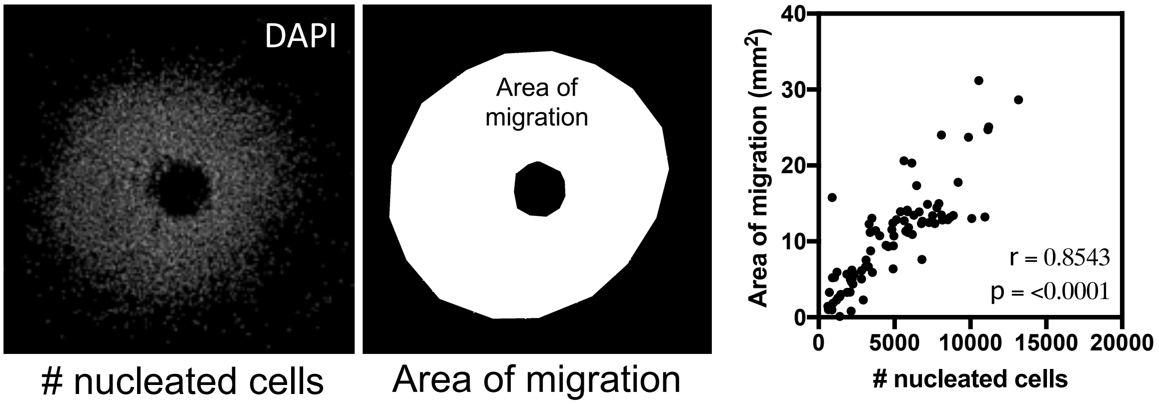

Figure S10. The extent of migration of the hiPSC-derived cell population (here quantified by the surface area of the substratum covered by the population of nucleated cells, DAPI+, external to the spheroid) is positively correlated with the number of cells generated from a spheroid. R is the Pearson coefficient; n = 83 spheroids pooled from two independent experiments with lines two and five; $p < 0.0001$.

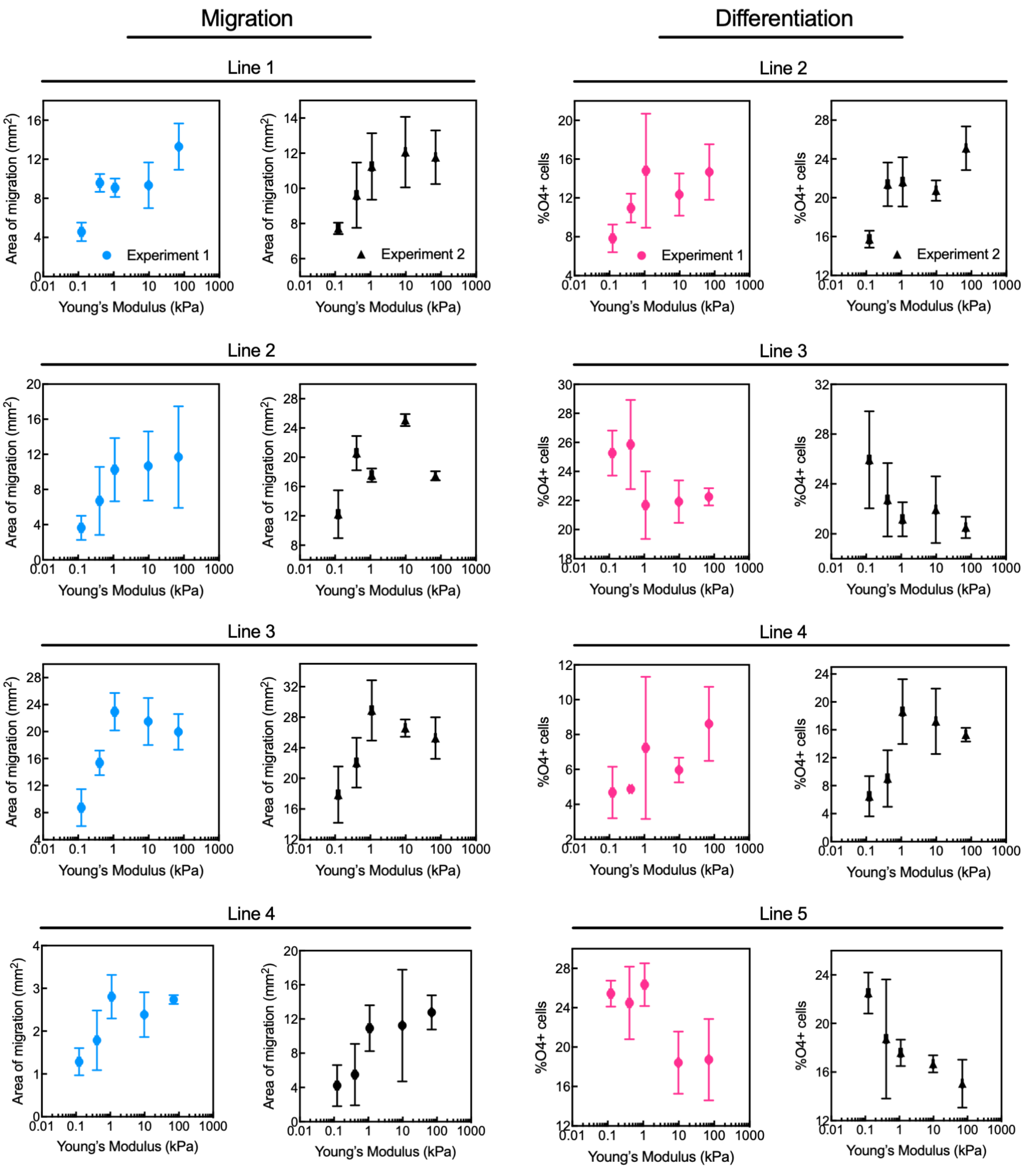

Figure S11. Area of migration (from Figure 2B) and % O4+ cells (from Figure 3B) with y axes adjusted to the data range of each independent experiment.

Supplementary methods

Rat oligodendrocyte differentiation (Figure S9)

Oligodendrocyte progenitor cells (OPCs) were isolated from mixed cultures of Sprague Dawley rats (BioreclamationIVT), as described previously (Jagielska et al., 2012). Polyacrylamide hydrogels were prepared and functionalized with poly-L-ornithine and laminin as described in the Methods section of the main article. Tissue culture polystyrene (TCP) plates were functionalized with poly-L-ornithine and laminin as described in the Methods section of the main article. OPCs were plated on hydrogels and TCP at a density of 2,500 cells/cm^2, and maintained in progenitor state for 24 hours in DMEM with SATO modification (5 mg/mL insulin, 50 mg/mL holo-Transferrin, 5 ng/mL sodium selenate, 16.1 mg/mL putrescine, 62 ng/mL progesterone, and 0.1 mg/mL bovine serum albu- min), 10 ng/mL platelet-derived growth factor homodimer AA (PDGF-AA, Peprotech) and 10 ng/mL basic fibro- blast growth factor-2 (FGF-2, Peprotech) (proliferation medium). Differentiation was induced after 24 hours in SATO's medium with 0.5% FBS, without PDGF-AA and FGF-2 (differentiation medium). Cells were fixed and immunostained after 6 days in differentiation medium. Live cells were incubated in differentiation medium containing 5% goat serum for 15 min at 37°C; followed by incubation with O4 antibody in differentiation medium containing 5% goat serum for 45 min at 37°C; and washed twice with DMEM and once with PBS. Cells were immediately fixed with 4% paraformaldehyde in PBS for 20 min, washed three times with PBS, and permeabilized with 0.2% Triton X-100 for 5 min. Permeabilized cells were washed with PBS three times and blocked with 5% goat serum in PBS for one hour at room temperature; followed by overnight incubation with primary rat anti-MBP antibody (Table S9) in 5% goat serum at 4°C; washed three times with PBS and incubated with secondary antibodies (Alexa Fluor, Invitrogen) at 1:500 dilution in PBS, protected from light. After three washes with PBS, nuclei were stained with Hoechst 33342 (Thermo Fisher, cat. no. H3570) at 1:1000 dilution in PBS for 5 min. Images were acquired at 10x magnification with an inverted Olympus IX-81 microscope and analyzed as described in the Methods section of the main article.

References

Jagielska, A. et al. Mechanical environment modulates biological properties of oligodendrocyte progenitor cells. Stem Cells Dev. 21, 2905–2914 (2012).
